# Home Environment as a Therapeutic Target for Prevention and Treatment of Chronic Diseases: Delivering Restorative Living Spaces, Patient Education and Self-Care by Bridging Biophilic Design, E-Commerce and Digital Health Technologies

**DOI:** 10.3390/ijerph22020225

**Published:** 2025-02-05

**Authors:** Dorothy Day Huntsman, Grzegorz Bulaj

**Affiliations:** 1 Dayhouse Studio, Salt Lake City, UT 84106, USA; dorothy@dayhousestudio.com; 2 OMNI Self-Care, LLC, Salt Lake City, UT 84106, USA; 3 Department of Medicinal Chemistry, College of Pharmacy, University of Utah, Salt Lake City, UT 84112, USA

**Keywords:** biophilia, built environment, mHealth, PDURS, AI, health at home, hospital at home, home care, lifestyle medicine, self-management

## Abstract

A high prevalence of chronic diseases exposes diverse healthcare pain points due to the limited effectiveness of pharmaceutical drugs and biologics, sedentary lifestyles, insufficient health literacy, chronic stress, unsatisfactory patient experience, environmental pollution and competition with commercial determinants of health. To improve patient care and long-term outcomes, the impact of the home environment is overlooked and underutilized by healthcare. This cross-disciplinary work describes perspectives on (1) the home environment as a therapeutic target for the prevention and treatment of chronic diseases and (2) transforming health-centric household goods e-commerce platforms into digital health interventions. We provide a rationale for creating therapeutic home environments grounded in biophilic design (multisensory, environmental enrichment) and supporting physical activities, quality sleep, nutrition, music, stress reduction, self-efficacy, social support and health education, hence providing clinical benefits through the modulation of the autonomic nervous system, neuroplasticity and behavior change. These pleiotropic “active non-pharmacological ingredients” can be personalized for people living with depression, anxiety, migraine, chronic pain, cancer, cardiovascular and other conditions. We discuss prospects for integrating e-commerce with digital health platforms to create “therapeutic home environment” interventions delivered through digital therapeutics and their combinations with prescription drugs. This multimodal approach can enhance patient engagement while bridging consumer spending with healthcare outcomes.

## 1. Introduction

Approximately half of the adults living in the United States (US) have at least one chronic disease [1]. For example, among US adults, 51.6 million experience chronic pain (2021 data [2]), 47 million have been diagnosed with depression (2020 data [3]), 40 million have migraine (2018 data [4]) and 16.9 million have anxiety (2018 data [5]). It is estimated that approx. 18.1 million US cancer survivors live as of 1 January 2022 [6]. Each person living with a chronic disease experiences a diminished health-related quality of life (HRQoL) due to symptoms that impact daily activities, which can also lead to disabilities. Chronic diseases negatively impact patients’ families (financial and emotional burdens), along with healthcare professionals (burnout) and healthcare systems (increasing healthcare costs).

In addition to impacting individuals and public health, the high prevalence and incidence of chronic diseases contribute to increasing healthcare spending in the US (USD 4.9 trillion in 2023, or 17.6% of the nation’s gross domestic product, GDP) [7]. The study from the Milken Institute estimated that the costs of direct healthcare treatments of chronic diseases in the US were USD 1.1 trillion in 2016 [8]. It is estimated that a macroeconomic burden of all non-communicable chronic diseases (NCDs) in the US could reach a total of USD 94 trillion for the period 2015–2050 [9].

The prevention and treatment of chronic diseases pose multiple challenges to healthcare professionals and systems. A combination of a patient’s lifestyle behaviors and DNA polymorphism (genetic diversity) impact the effectiveness of pharmacological interventions. Adverse effects of prescription drugs, medical errors, healthcare accessibility and affordability contribute to increased morbidity and mortality. Environmental pollution and commercial determinants of health negatively impact population health [10,11,12,13]. Examples of diverse pain points associated with medicine and healthcare outcomes in the US are illustrated in Table 1.

To reduce the prevalence of chronic diseases, some possible solutions include (1) early detection, (2) scaling up lifestyle medicine through digital health technologies and (3) public health interventions through health promotion and education. These solutions are supported by the growing availability of wearables that enable monitoring vital signs and health-centric behaviors, advances in artificial intelligence (AI) and machine learning (ML) and the validation of digital biomarkers that contribute to the early detection of medical conditions [50,51,52,53]. Digital health technologies also enable the integration of behavioral interventions and lifestyle medicines with pharmacotherapies [54,55,56]. On the other hand, barriers to scaling up interventions for chronic diseases include (1) misaligned funding for biomedical research and public health interventions disproportionally favoring innovations of treatments rather than prevention [57,58,59], (2) poor health literacy [60,61] and (3) inadequate implementation of evidence-based practices to prevent chronic diseases in community settings [62].

The impact of the home environment on a person’s health has been underutilized in healthcare, even though the built environment can improve health outcomes [63,64,65,66,67]. Housing–health relationships are well characterized, including prospective interventional studies on how internal housing conditions can improve health outcomes [23,24,68,69,70,71]. Smart home technologies further illustrate opportunities to create at-home healthcare applications [72,73,74]. Our previous work described the biophilic design of home environments, fostering disease-specific self-care for people living with migraine, chronic pain and depression [22]. Herein, we spotlight the perspectives to harness 125 million households in the US (2018–2022 data from the US Census Bureau) to deliver evidence-based solutions for the prevention and adjunctive treatment of diverse chronic diseases via a health-centric e-commerce platform.

**Figure 1:**
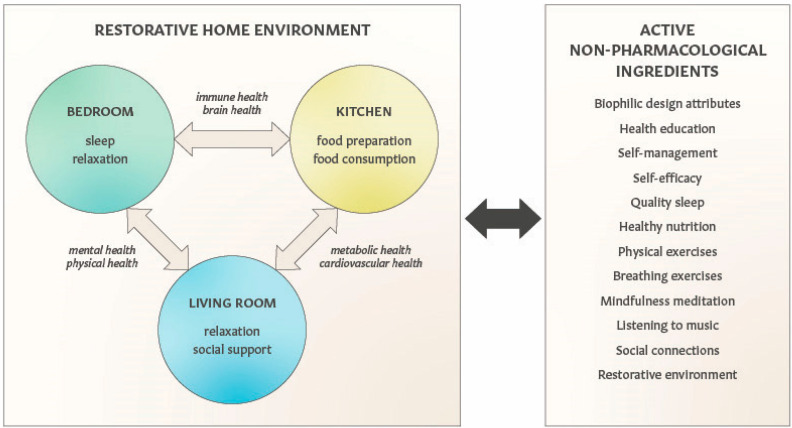
The relationships between the home environment, self-care and health. A combination of daily self-care activities, a restorative home environment, patient education and a reduced exposure to indoor environmental pollution can positively impact physiological functions and health outcomes of household occupants.

Examples of home–health connections and their relationships with self-care are illustrated in Figure 1. There is growing evidence that diverse non-pharmacological modalities and self-care practices elicit clinically meaningful benefits and can also reduce the risks of chronic diseases. Perhaps the most commonly appreciated therapeutic modalities that exert pleiotropic effects on mental and physical health are (1) physical activities [75], (2) nutrition [76], (3) quality sleep [77], (4) music [78], (5) mindfulness practices [79], (6) social connections [80] and (7) exposure to nature [81]. In clinical practice guidelines, the American College of Physicians recommends exercise, mindfulness-based stress reduction and yoga as the first line of therapy for chronic low back pain [82]. However, integrating these modalities as “active non-pharmacological ingredients” into patient care poses multiple challenges ranging from feasibility and clinical validation to regulatory requirements and implementation by healthcare and households. To overcome these challenges and catalyze a broader adoption of multimodal interventions, we describe (1) the home environment as the therapeutic target for the prevention and adjunctive treatment of diverse chronic diseases, (2) a household goods e-commerce platform as the delivery system for biophilic interventions, evidence-based self-care and health education and (3) the transformation of such health-centric e-commerce platforms into digital health interventions that can be further integrated with pharmaceutical drugs and biologics for better therapy outcomes.

This perspective article addresses the research gap related to the role of the home environment in improving the treatment and prevention of chronic diseases. The research on lifestyle interventions is usually limited to patients’ behavior change while generally excluding the context of their home environment [24]. Given that people in the US spend on average over 17 h/day at home (pre-COVID-19 pandemic data, and over 18 h/day in 2022 [83]), the long-term impact of the home environment on health outcomes has been overlooked in the research and underutilized in the healthcare environment.

This work also aims to overcome several limitations in existing approaches to the treatment and prevention of non-communicable chronic diseases. For example, the limited effectiveness of pharmaceutical drugs and biologics can be attributed to factors such as medication non-adherence, adverse effects, toxicity and drug resistance. Similarly, digital therapeutics face challenges such as maintaining patient engagement, limited digital health literacy and long-term behavior change. Moreover, once patients receive treatments that target disease symptoms and underlying causes, they often lack a continuum of care with respect to the tertiary prevention after reaching remission. The prevention challenges are further compounded in real-world circumstances, where patients are continuously exposed to the commercial determinants of health that compete with healthy lifestyles. Lastly, this perspective tackles the research–practice gap in integrating lifestyle medicine and prevention with daily activities of people living with or at risk of chronic diseases.

## 2. Home Environment as a Therapeutic Target for the Prevention and Treatment of Chronic Diseases

**Figure 2:**
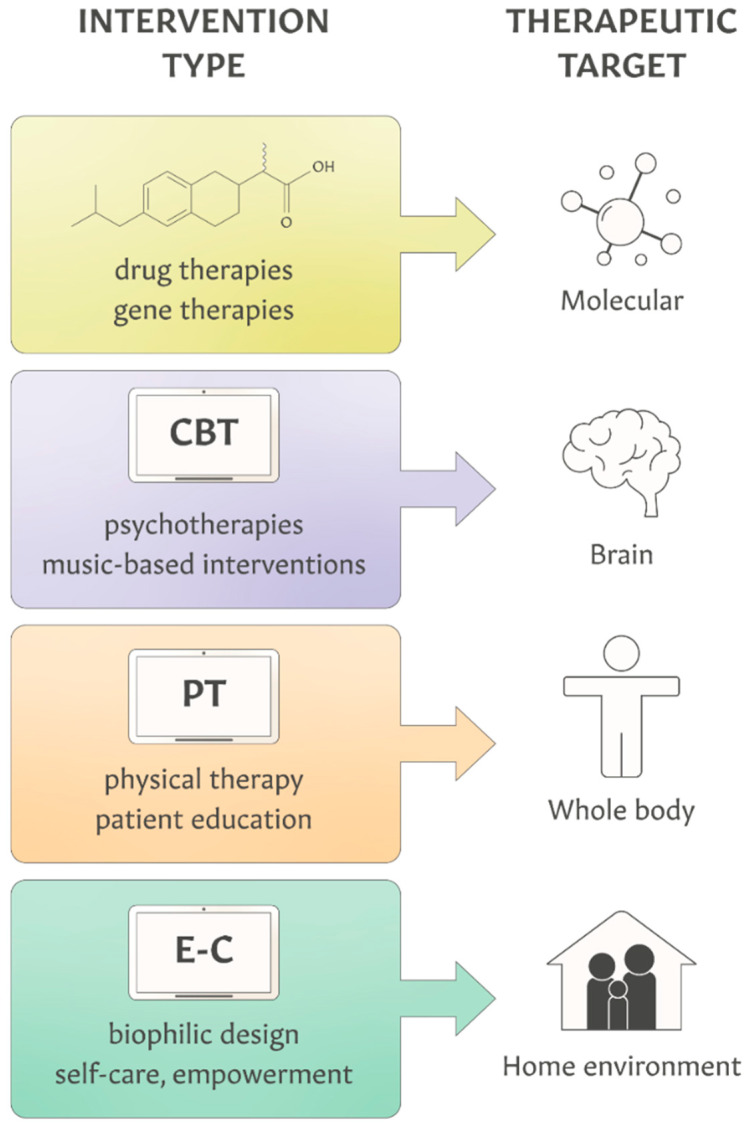
A comparison of diverse categories of therapeutic targets for the prevention and treatment of chronic diseases. A mobile device illustrates an ability to deliver therapies through digital health technologies, including digital therapeutics (DTx) and “prescription drug use-related software” (PDURS) framework that enables the integration of DTx with pharmacotherapies. CBT—cognitive behavioral therapy; PT—physical therapy; E-C—e-commerce.

Treatments for chronic diseases such as pharmacological, behavioral and physical therapy interventions are traditionally focused on engaging a patient’s molecular targets (e.g., proteins, DNA, metabolites), human body systems (e.g., the brain, the autonomic nervous system) or the whole human body to elicit specific physiological responses and clinically meaningful benefits (Figure 2). These patient-centric interventions have been developed and validated via randomized clinical trials (RCTs) and have undergone rigorous evidence-based reviews before being implemented into medical practice. While helping with disease management and remission, these therapeutic modalities have limited effectiveness due to patients’ non-compliance, genetic and epigenetic polymorphisms, drug adverse effects and toxicity, unhealthy lifestyles, chronic stress and environmental pollution.

A growing interest in harnessing the home environment and self-care to improve healthcare outcomes can be illustrated by hospital-at-home and disease self-management programs aimed to innovate acute care delivery, reduce healthcare costs and improve therapy outcomes [84,85,86]. Promoting healthy home environments and lifestyles has been effective in improving physical activity, nutrition and self-efficacy to prevent obesity [87]. Home modifications are recognized as a means to prevent physical injuries [88]. It has been proposed that the judicious design of a home environment can improve symptoms for people living with diverse chronic conditions [22,89,90]. Home-based interventions for chronic conditions also include therapies delivered via digital health technologies [91,92,93].

A home environment, as defined here, encompasses the physical, chemical and emotional aspects of a housing unit along with its occupants who share daily activities and experiences. The compounding effects of the indoor living space, outdoor surroundings, daily habits and interpersonal relationships can either positively or negatively impact chronic illness [94,95,96,97]. To the best of our knowledge, the home environment has not been proposed as a therapeutic target *intended* for the prevention (primary and tertiary) or the adjunctive treatment of specific medical conditions (Figure 2). For example, a combination of biophilic interior design, listening to music, physical exercises and self-help can improve emotional health and reduce symptoms of anxiety and depression [66,98,99,100,101,102,103]. Because of its complexity, the home environment can be considered as a multimodal therapy target, eliciting pleiotropic effects through a combination of anti-inflammatory effects, the modulation of the autonomic nervous system, neuroplasticity and behavior change (mechanisms of action are discussed later).

## 3. Impact of Biophilic Interior Design, Self-Care, Patient Education and Environmental Pollution on Health Outcomes

The built environment can either improve or worsen the health and well-being of its occupants [67,104,105,106,107,108]. Improving health and well-being through interior design, architecture and urban planning has been recognized and practiced for many years [63,65,109,110,111]. The main goal of interior design is to provide functionality, safety, comfort and esthetically enriching experiences. Connections between interior design, architecture and health [63,112] have been reflected in the design of hospitals [65,90,113,114]. Optimizing the design of the indoor environment to maximize esthetic experiences and emotional responses can support brain health [115,116]. Moreover, the study of urban landscaping design reported a “positive correlation between perceived attractiveness and restorative effect” [117], while another study showed correlations between the perceived esthetics of neighborhoods and the mental well-being of their residents [118].

Biophilic design connects the built environment and its occupants with nature through multisensory experiences [119,120], and its beneficial effects have been demonstrated in healthcare, educational, hospitality, office and other commercial spaces [22,64,65,90,101,120,121,122,123,124,125,126]. A systematic review of the healthcare outcomes of biophilic design reported that “biophilic design in hospitals reduces hospitalization time, patient mortality, pain levels, and stress for healthcare providers. It alleviates anxiety, improves experiences for patients, families, and staff, reduces patient harm, and supports faster recovery” [109]. Restorative properties of biophilic design and exposure to indoor nature elicit the following responses: (1) modulation of the autonomic nervous system [127,128,129], (2) stress reduction [130,131], (3) increased relaxation [129,132,133], (4) increased positive emotions and reduced negative affect [127,134], (4) reduced risks of depression and anxiety [135], (5) reduced blood pressure [136,137], (6) lowered heart rate [127,138], (7) calming of the prefrontal cortex brain activity [133], (8) improved cognitive functions [138,139,140], improved pain relief and postsurgical recovery [136] and modulation of immune functions [141]. While the quality of research evidence varies for the aforementioned health benefits of biophilic design, these studies suggest that biophilic environments (nature-based environmental enrichment) can positively impact mental and physical health and alleviate disease symptoms for several chronic conditions [66,101,142].

Examples of household goods important for creating biophilic and health-centric spaces include (1) smart lighting systems that provide dimmable natural light supporting circadian rhythms and quality sleep, (2) furniture made of natural materials that minimize exposure to harmful chemicals, (3) rugs and window coverings made of natural materials for enhancing visual and haptic experiences in spaces that foster relaxation, (4) clay planters for indoor plants to reduce stress, blood pressure and improve positive mood and (5) air quality equipment including air quality monitoring systems and air purifiers to create air flow and mitigate indoor air pollution. Additionally, comfortable clothing made from natural materials are already considered an interior design attribute that can improve mood and relaxation [143,144].

Creating home environments intended to improve health outcomes also involves reducing exposure to health-harming hazards. The unhealthy environment, including chemical and physical pollution, is a known contributor to chronic diseases, including cancer and cardiovascular, respiratory, mental, neurological and neurodegenerative disorders [21,145]. Air and drinking water pollution is a public health issue in the US [146,147]. Chronic exposure to air pollution at home can cause adverse health effects [148,149,150,151]. Table 2 lists examples of chemical and physical factors that negatively impact overall health, exacerbate disease symptoms and may serve as underlying etiologies of chronic conditions. Reducing the exposure of household residents to environmental pollution at home can be used as the primary or/and tertiary prevention strategy for chronic conditions and comorbidities before and after remission. For example, for cancer survivors, it may be beneficial to use personal care products that are free from endocrine-disruptive chemicals and to reduce exposure to noise and light pollution that may disrupt circadian rhythms and quality of sleep and thus negatively impact the immune system and mental health.

Self-care is defined as “activities individuals undertake in promoting their own health, preventing their own disease, limiting their own illness, and restoring their own health” [170] or, according to the World Health Organization, “the ability of individuals, families and communities to promote health, prevent disease, maintain health, and to cope with illness with or without the support of a healthcare provider”. While self-care and self-management are sometimes used interchangeably, self-care has a broader meaning, since it encompasses self-management and self-efficacy [171]. The effectiveness of self-care as a means to prevent depression, dementia and stroke emphasizes its value for reducing the prevalence of chronic conditions [172,173].

In our previous works, we provided examples of evidence-based self-care practices that offer clinical benefits for people living with depression, chronic pain, migraine, epilepsy and cancer [22,54,56]. Self-management for arthritis, diabetes, cardiovascular conditions and mental health include symptom and stress management, relaxation techniques, nutrition, exercise and medication management [86]. There is also growing evidence that self-management yields clinical benefits in cardiovascular and metabolic diseases [174], mental disorders [175,176], chronic kidney disease [177], chronic low back pain [178], arthritis [179] and cancer [180]. Chronic disease self-management programs have been recognized as a means to reduce healthcare utilization [85,181].

While most laypeople and healthcare professionals are aware of the health benefits of physical activity and nutrition, an evidence–practice gap persists for these and other self-care modalities. For example, few people are aware of the importance of quality sleep for improving immune functions and reducing inflammation [182,183] or that listening to music can (1) reduce epileptic seizures [184,185,186], (2) produce analgesic effects in chronic pain conditions [187,188,189], (2) reduce depressive symptoms [99,100,190], (4) enhance immune functions [191,192] and (5) provide clinical benefits for people with Parkinson’s disease, multiple sclerosis and for those recovering from stroke [78,193,194].

Patient education and empowerment can also be considered as “active non-pharmacological ingredients” for improving therapy outcomes. Patient empowerment is a healthcare paradigm aimed at improving self-determination and a person’s locus of control (the perception of the ability to influence life’s circumstances) [195]. Patient education has been recognized as a means to improve pain management [196,197], reduce symptoms of rheumatoid arthritis [198] and decrease fatigue, depression, anxiety and pain in cancer patients [199]. Patient education positively impacts health literacy, which is important for improving outcomes and preventing chronic diseases [200,201,202]. Strategies for health education and dissemination of health-related information are diverse and include web-based programs, social media, mobile apps or digital marketing.

## 4. Designing Therapeutic Home Spaces for Specific Chronic Conditions

The therapeutic home environment approach described here is based on several theoretical frameworks related to health, ecosystems and environmental psychology. For example, the nature-based biopsychosocial resilience theory, attention-restoration theory and biophilia hypothesis focus on the importance of connections with natural environments [203,204,205]. Grounded in the place attachment theory, the person/process/place framework describes relationships between physical spaces, experiences and emotional and cognitive responses [206,207]. Ecological models of health behavior emphasize the effectiveness of multilevel interventions, including the home environment and household activities [208], while health behavior theories underline a person’s beliefs, cues and self-efficacy as key factors determining behavior change [209]. An underlying principle of therapeutic home environments is that long-term improvements of health outcomes for household residents depend on the continuous fostering of their restorative state through biophilic home spaces and self-care activities grounded in lifestyle medicine [22,97,210,211,212].

The applications of biophilic spaces that support disease-specific self-care were described previously [22], and combining environmental and behavioral interventions for better health outcomes is not an original concept [213]. Designing restorative spaces is a prerequisite for creating effective therapeutic home environments, as they support both mental and physiological recovery from health-harming stressors [214]. By emulating the restorative properties of the natural environment, biophilic interior design serves as an evidence-based strategy to promote relaxation and homeostatic rebalancing of the autonomic nervous system [127,215,216,217]. Below, we outline the perspectives for developing “therapeutic home environment” interventions aimed at improving the prevention and treatment of diverse chronic diseases.

For people living with or who are at risk of mental disorders, biophilic home environments offer a place for comfort, refuge and feeling safe. The experience of nature is known to improve mood and mental health [81,218,219,220]. Research suggests that even a view of nature from a window can reduce the risk of depression and anxiety [135]; furthermore, exposure to natural sounds can reduce anxiety, stress and annoyance while increasing positive emotions [221,222]. The quantity and quality of natural spaces can decrease mental healthcare utilization, including outpatient visits for those living with depression and bipolar disorder [223,224]. Designing therapeutic home environments for people with depression aims at increasing exposure to natural light, incorporating indoor elements that promote positive emotions and arousal through biophilic design and multisensory experiences [22].

**Figure 3:**
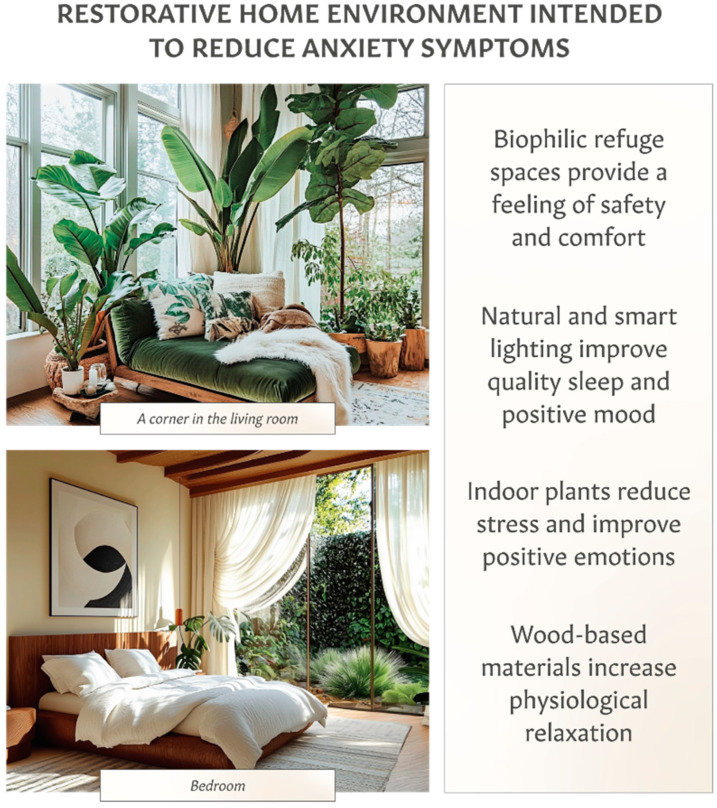
Therapeutic home environments for people living with anxiety. Specific design features create multisensory experiences intended to provide restorative effects, increase relaxation and activate the parasympathetic nervous system. Unseen elements of the therapeutic home environment include personalized soundscapes (including ambient music), scentscapes and patient education delivered through an integrated household goods e-commerce and digital health platform.

For anxiety, biophilic elements improve relaxation, as reflected in reducing sympathetic and increasing parasympathetic activity [127,215,216,217]. As illustrated in Figure 3, creating a refuge space at home that incorporates house plants and wooden elements, like furniture and flooring, can further enhance relaxation effects through both visual and non-visual perceptions [133,225,226,227,228,229]. A refuge space fosters the feeling of safety, leading to stress reduction and the restorative state [210]. Additional examples of biophilic interior designs for people living with anxiety are shown in Figures S1 and S2 (Supplementary Materials). Given the sleep–anxiety relationship, it is important to optimize the bedroom to simultaneously improve sleep quality and reduce anxiety symptoms [230]. For example, research shows that the quality of sleep is improved in a bed made of solid wood compared with one made of melamine panels designed to look like wood [229]. A multisensory biophilic intervention to reduce anxiety symptoms can also include aromatherapy with essential oils [231]. For self-care practices to reduce anxiety symptoms at home, clinicians recommend physical exercises [232].

For neurological disorders, such as chronic pain or migraine, therapeutic home environments offer a means to activate the parasympathetic nervous system through biophilic design, which acts as a neuromodulation of the autonomic nervous system. These patients can benefit from a personalized multisensory space that fosters relaxation techniques, other self-care practices and lifestyle medicine for pain relief or the reduction of migraine headaches [22,233,234,235]. Optimized lighting systems can serve as photobiomodulation interventions for people living with migraine or fibromyalgia [236,237,238]. For people with epilepsy, a combination of a restorative home environment and listening to specific music compositions can improve seizure control [184,185,239,240]. Multisensory experiences delivered through a biophilic home design and exposure to the outdoor natural environment can support mental, cognitive and physical functions for people with dementia, Alzheimer’s and Parkinson’s disease [89,90,134,241,242,243,244].

Natural environments elicit therapeutic benefits for cardiovascular disorders, such as the reduction of blood pressure [134,137] and heart rate [245,246]. A biophilic home environment can reduce stress that contributes to the risk and severity of hypertension and coronary heart disease [247,248]. In addition to stress reduction, lifestyle changes associated with physical activities, sleep and nutrition can improve markers for cardiovascular and cardiometabolic risks [249,250]. The stress-reducing benefits of biophilic spaces are applicable to the management of diabetes and obesity [251,252].

For oncology patients, biophilic design is important to create both clinical and non-clinical therapeutic environments [253]. Cancer survivors are also encouraged to integrate self-management into their daily activities [254]. Self-managing mental and physical conditions (e.g., fatigue, pain, anxiety, depression, self-confidence) includes healthy lifestyle behaviors and self-care practices, along with self-determination, social support, medication management, education and communications with care providers [199,255,256,257]. Since both chronic stress and chemotherapy can weaken immune functions, thus impacting cancer prognosis [258,259], it is equally important to create a biophilic and restorative home environment that fosters self-care practices to enhance immune functions. Examples of this would be listening to music [191,260,261], physical exercises [262,263], healthy nutrition [264,265] and quality sleep [182,266].

Although biophilic design is recognized by healthcare as a promising means to improve patients’ experience and outcomes, it has been underutilized to create health-centric homes and living communities. Barriers to a broader adoption of biophilic design in residential spaces include (1) a lack of awareness about the health benefits of biophilic spaces among the general population and (2) a lack of scalable delivery methods of biophilic interventions that can be integrated with the healthcare ecosystem. Since digital health technologies have rapidly grown over the past decade, they are positioned to integrate biophilic and behavioral interventions. The delivery and scalability of such integrated therapies can be accomplished by transforming health-centric household goods e-commerce platforms (software as a service, SaaS) into digital therapies (software as a medical device, SaMD), as described below.

## 5. The Delivery of Therapeutic Home Environments Through the Integration of e-Commerce and Digital Health Platforms

**Figure 4:**
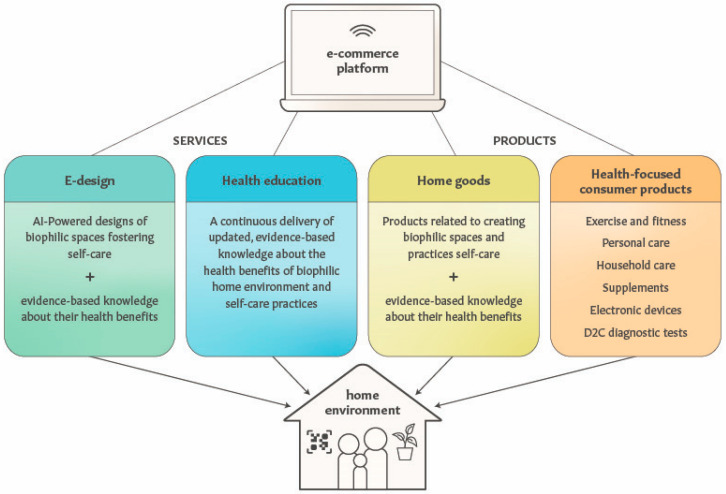
An e-commerce platform as the delivery system for health-centric services and products to create and sustain therapeutic home environments and behavior change. QR code symbolizes technologies as the enablers of health education for household occupants.

An increasing number of people use e-commerce for their daily shopping needs [267,268,269], while the evidence of software-based solutions for improving lifestyle modifications and healthcare outcomes is also increasing [270,271]. There are numerous household goods that align with biophilic design, diverse self-care practices and healthy home environments. When these goods are available via online shopping together with a description of their associations with health-centric behaviors and the potential impact on health outcomes, such an e-commerce platform can serve as the delivery system for consumer products and education combined. The high prevalence of chronic diseases and consumer-driven economy in the US offer timely opportunities to integrate health-centric e-commerce and digital health platforms to target home environments as prevention and adjunctive therapies for people living with or at risk of chronic conditions. As illustrated in Figure 4, a health-centric e-commerce platform could deliver diverse categories of products, services and information that, collectively, may lead to improving disease prevention and prognosis.

The delivery of health-focused designs, products and patient education is a prerequisite for transforming the home into a therapeutic environment. Virtual reality and AI-powered software solutions are increasingly used in interior design and architecture [272,273,274], enabling the users to create and visualize virtual spaces. Such e-design services, offered through e-commerce platforms, would help users to optimize their home environment while providing science-informed descriptions of potential health benefits of specific layouts and household goods. Rapid advances in AI are facilitating the development and applications of e-commerce platforms [275]. While biophilic design offers a means to bring the health benefits of nature, it is health education that provides the understanding of how exposure to nature can positively impact health.

**Figure 5:**
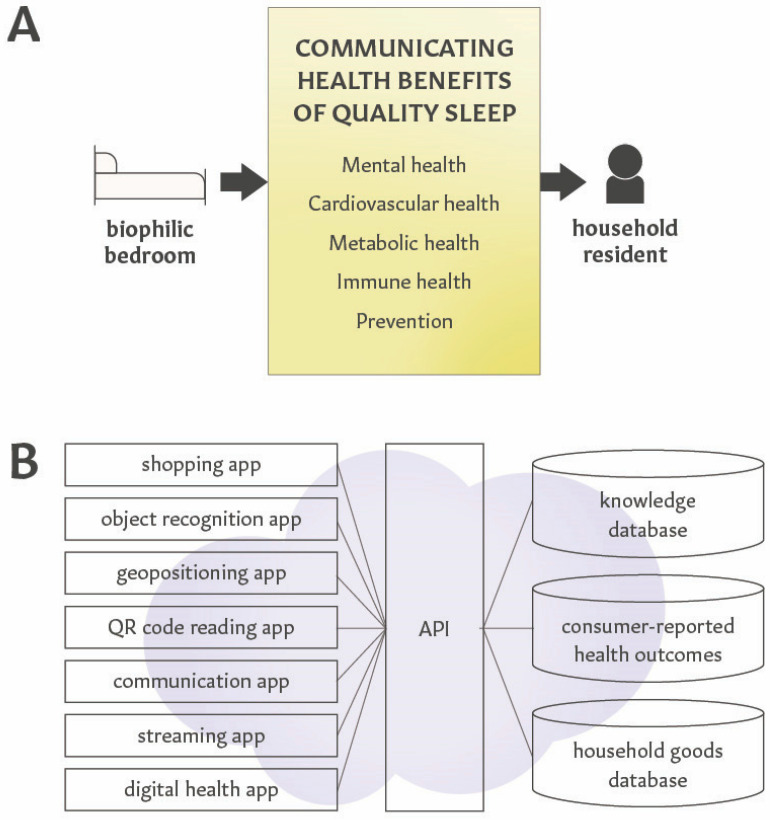
Personalized communications between health-centric home environment and its occupants. (**A**) An example of how a biophilic bedroom environment and its individual components can communicate evidence-based knowledge and updates on the impact of quality sleep for specific health conditions. (**B**) An example of a patent-pending infrastructure providing personalized information to home occupants about the health benefits of specific household items fostering self-care practices.

In addition to using household goods for creating health-centric home environments, the functionality of these goods can be expanded by using them for patient education. Patient-reported health outcomes can inform the e-commerce platform to personalize data-driven interventions by adjusting health education content for a patient’s specific needs. As illustrated in Figure 5, a bed located in the bedroom can be used to deliver personalized health education messages about the relationships between sleep and mental or cardiovascular health, or chronic pain and migraine headaches, depending on the consumer’s health condition. Similarly, by integrating object recognition and communication apps, the same piece of furniture (e.g., dining or kitchen table) or tableware can be used to disseminate tailored education and recommendations, such as nutrition guidance for health-centric meal preparations that are customized to household residents [276]. Recent advances in AI-powered chatbot interventions illustrate perspectives in combining personalized health education with virtual health coaching and disease management that utilize feedback from patient-reported outcomes and data generated by wearable devices (e.g., sleep patterns, physical activity, stress levels, continuous glucose monitoring, disease symptoms) [277,278].

Such personalized and on-demand health education can be provided by e-commerce platforms by using application programming interfaces (APIs) that integrate diverse communication technologies [279]. Figure 5B illustrates an example of infrastructure that integrates a home-centric e-commerce platform with personalized health education. Health education can also be delivered visually or acoustically through consumer electronics, such as mobile devices, or the smart Frame TV, which has been previously described as the delivery system for biophilic multisensory interventions [22]. Similarly, applications of QR codes for health promotion and education were reported before [280,281]. The household occupants’ awareness of their instant access to evidence-based knowledge and personalized updates about their health-centric home environment serves as an empowerment tool to sustain behavior changes.

Many consumer products, e.g., wearable fitness trackers, are recognized for their abilities to improve health outcomes while also empowering lifestyle behaviors related to prevention and treatment of chronic diseases, including depression, anxiety, obesity and hypertension [282,283,284,285,286]. Examples of diverse categories of consumer goods that can impact health outcomes are (1) exercise and fitness equipment [287,288], (2) personal and household care products that minimize exposure to hazardous chemicals [289,290], (3) supplements that produce clinically meaningful effects, e.g., St John’s Wort for depression [291], (4) direct-to-consumer in vitro diagnostic tests [292] and (5) educational materials supporting evidence-based self-care [86,293]. Such products, delivered via e-commerce platforms, are paired with the user experience tailored to specific health needs, supporting the creation of a therapeutic home environment.

The intention to treat and prevent chronic diseases by therapeutic home environments that integrate household goods e-commerce, biophilic design, healthy home interiors, self-care and personalized health education is a novel approach to improve healthcare outcomes. When creating therapeutic home environments, a multimodal combination of restorative spaces and personalized “active non-pharmacological ingredients” can lead to clinically meaningful benefits for all household occupants. Such an integrative approach can also be defined as a digital/behavioral/environmental intervention yielding pleiotropic effects through diverse mechanisms of action, as shown in Table 3. The combination of nature-based experiences and self-care practices at home enables the modulation of the autonomic nervous system and neuroplasticity through diverse mechanisms. For example, biophilic interventions were shown to affect autonomic functions through stress reduction, an accelerated recovery from stress and enhanced relaxation, while environmental enrichment is well documented to stimulate neuroplasticity [294]. Housing environmental enrichment was proposed as a means to modulate neurogenesis [295]. Similarly, lifestyle interventions and self-care, including physical activities, music, quality sleep, nutrition and education, can cumulatively impact the autonomic control by modulating the stress response and neuroplasticity through cognitive engagement and modulating expression of brain-derived neurotropic factors [296,297,298,299,300].

Digital health technologies accommodate diverse non-pharmacological modalities that are used for health promotion, prevention and the management of chronic disease [309,310,311,312,313] (also illustrated in Figure S3, Supplementary Materials). They are also helpful tools for motivational and just-in-time adaptive interventions supporting lifestyle modification [314]. Digital health technologies show promise for the hospital at home programs and geriatric care [315,316,317,318]. An unprecedented expansion of software-based solutions to improve health outcomes include “software as a medical device” products (also known as digital therapeutics, or DTx) intended to diagnose and treat medical conditions, and a “prescription drug use-related software” (PDURS) framework allowing the integration of mobile apps with specific pharmaceutical drugs and biologics [55,319,320]. Since many digital health technologies are considered low risk to patients, they can be marketed under the FDA’s enforcement discretion [321,322].

**Figure 6:**
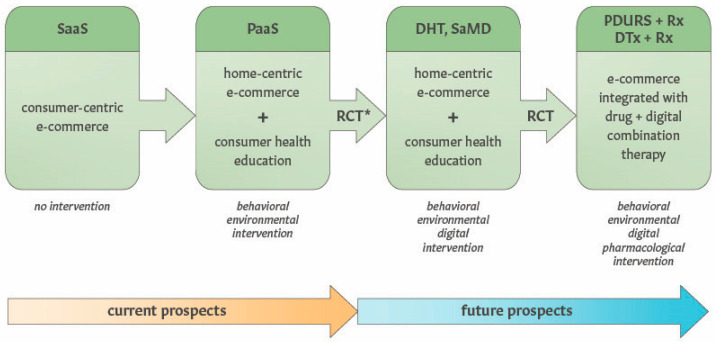
Perspectives on transforming e-commerce platforms into digital/behavioral/environmental combination therapies for chronic disorders. Integration of e-commerce and the therapeutic home environment with pharmacological treatments can be accomplished through adjunctive digital therapeutics or a “prescription drug use-related software” framework. RCT—randomized control trial; PaaS—platform as a service; DHT—digital health technology, SaMD—software as a medical device; PDURS—“prescription drug use-related software” framework; DTx—digital therapeutic. * denotes that the feasibility testing can involve prospective observational studies.

While traditional e-commerce platforms are not intended to treat or prevent chronic diseases, we describe the perspectives for transforming e-commerce into digital health technology that can deliver behavioral/environmental combination therapies (Figure 6 and Figure S4). Implementing such a therapeutic strategy and business model requires validation of clinically meaningful benefits of the therapeutic home environment provided via the e-commerce platform (the delivery system for “active non-pharmacological ingredients”). The feasibility and real-world evidence for therapeutic applications of health-centric e-commerce platforms for people living with chronic conditions can be evaluated with prospective cohort studies. However, pivotal RCTs are required to determine the efficacy of software-based interventions in order to receive clearance or approval of marketing software for medical purposes.

As illustrated in Figure 6, long-term prospects for software-delivered “therapeutic home environment” interventions include the development of combination therapies comprising digital, behavioral and environmental interventions by following the SaMD regulatory pathway. The development of e-commerce as a digital therapeutic can be advanced by either (1) expanding an existing e-commerce marketplace platform through adding therapeutic content (e.g., biophilic interventions, patient education) or (2) improving the efficacy and effectiveness of an existing digital health platform by adding e-commerce functionality. Furthermore, once reaching the status of digital health technology, e-commerce platforms can be integrated with pharmacotherapies via either adjunctive DTx or the PDURS framework. Advancing digital + drug combination therapies using the health-centric e-commerce platform as the PDURS approach requires partnerships with pharma companies, which can also benefit by improving both the effectiveness of their blockbuster drugs and the market share after the loss of exclusivity [55].

Integrating digital health and e-commerce platforms into a multimodal “therapeutic home environment” intervention has the advantage of general applicability for the prevention and treatment of many chronic diseases, while simultaneously enabling personalized therapeutic content for specific chronic conditions using AI/ML [54]. The main differences between traditional digital health interventions and those described here are illustrated in Figures S4 and S5, Supplementary Material. In addition, the consolidation of digital interventions and consumer shopping can simultaneously improve patient engagement and health-centric consumer behavior. Ultimately, all stakeholders, including patients, caregivers, healthcare systems, pharma and digital health companies, consumer brands, investors and the general public will benefit from advancing “therapeutic home environment” interventions delivered by an e-commerce platform.

## 6. Unique Opportunities Meet Real-World Challenges

The integration of household goods e-commerce and digital health platforms to improve the prevention and treatment of chronic conditions offers a unique confluence of consumer spending and healthcare goals and provides win–win solutions for commercial and public health interests. It also mitigates barriers to implement lifestyle interventions [212,323]. “Therapeutic home environment” interventions using a digital health platform may also shorten the evidence–practice gap, since the FDA’s authorization of such multimodal digital interventions can seamlessly bridge diverse self-care practices and patient care.

**Figure 7:**
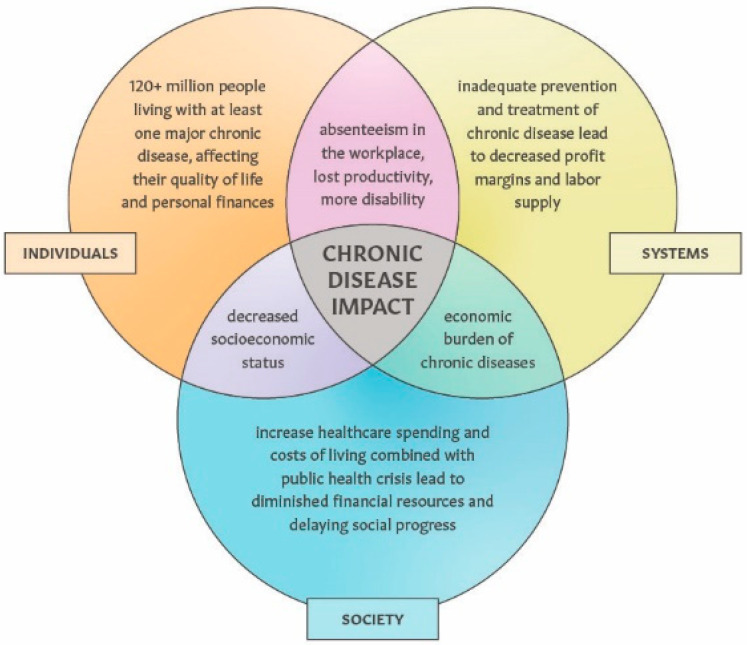
Overview of the impact and economic burden of chronic diseases on individual households, work places and society.

Creating a digital health platform delivering “therapeutic home environment” interventions conveys broader social and environmental impacts. As illustrated in Figure 7, a high prevalence of chronic diseases leads to a diminished health-related quality of life and a financial burden for many individuals, while also straining the workforce and society at large. Investing in innovative solutions to prevent non-communicable chronic diseases is less “attractive” compared with innovating ways to treat patients who suffer from illness [11,12,43,44]. This pain point is of particular importance in the context of insufficient public health spending to combat preventable chronic diseases in the US [59,324,325], while the incidence and prevalence of depression, anxiety, migraine cancer and other chronic conditions in younger populations is on the rise [326,327,328,329]. From a social impact perspective, scaling up prevention by building health-centric housing offers long-term solutions to reduce the overall burden of chronic diseases [330,331,332].

The environmental impact of integrating non-pharmacological modalities and behavior change to improve chronic disease prevention and treatments would likely reduce the considerable carbon footprint and chemical pollution associated with healthcare utilization and pharmaceutical industries [47,48,49]. Furthermore, using a household goods e-commerce platform to educate consumers about the health benefits of exposure to nature through biophilic design may foster partnerships between health insurance companies, urban planners and real estate developers to incorporate more neighborhood greenspaces, known to reduce healthcare utilization [224,333,334,335].

Adoption of an integrated e-commerce and digital health platform delivering therapeutic home environments can be appealing to multiple stakeholders, for example, healthcare systems, accountable care organizations, physician groups, telehealth, tech health and digital health companies, all of which can offer such e-commerce services to their members in order to improve patient experience, engagement, health literacy, self-efficacy and, ultimately, value-based care outcomes. For general wellness companies aiming to improve health and well-being for their customers, the integration of their platforms with health-centric e-commerce delivering therapeutic home environments may improve customer experience, retention and health outcomes while increasing the value proposition of these platforms. Figure S5 illustrates an example of using the B2B2C model to scale up the adoption of an integrated e-commerce and digital health platform delivering therapeutic home environments by diverse stakeholders.

For consumer brands, an e-commerce platform that associates their products with positive health outcomes for their customers can increase the value proposition, competitiveness and sales through health-centric marketing and strategic partnerships [336]. For pharmaceutical and biotech companies, applying PDURS or adjunct DTx frameworks to integrate their drug products with the therapeutic home environment through an e-commerce platform can enable (1) improvements of the effectiveness of their blockbuster drugs while mitigating the loss of market exclusivity [55], (2) the extension of their MOA through the modulation of the autonomic nervous system combined with the anti-inflammatory and neuroplasticity effects of the therapeutic home environment and (3) increasing patient engagement and satisfaction.

However, the prospects of using digital platforms as the delivery system for therapeutic home environments face a number of real-world limitations, including (1) a lack of strong evidence of clinically meaningful benefits of biophilic environments that demonstrate the long-term impact on chronic conditions, (2) a diverse quality of evidence of clinically meaningful benefits of specific self-care practices and their impact on chronic conditions [337] and (3) the impact of outdoor environmental pollution on human health. Examples of challenges for advancing therapeutic interventions targeting the home environment include (1) funding to improve research evidence on clinically meaningful benefits of biophilic and self-care interventions, (2) creating an e-commerce platform that maintains a digital therapeutics infrastructure including the protection of consumer/patient privacy information [338], (3) building trust between healthcare professionals and health-centric e-commerce, (4), evolving regulatory pathways that enable the integration of software with prescription drugs [55,322], (5) competing with the negative impacts of commercial determinants of health, (6) the long-term maintenance of healthy lifestyles while experiencing “only” incremental improvements in therapy outcomes [339], (7) the inequality in access to technology and the internet and (8) limited digital health literacy.

Implementation, adoption and scaling up “e-commerce as digital health” intervention requires addressing such challenges like patients’ privacy and data ownership concerns, cybersecurity risks, interoperability with healthcare IT infrastructure, AI-powered technologies, wearables and other medical devices, to name a few examples. Cyber threats to healthcare systems explore human and software vulnerabilities [340,341], which are also relevant to the integrated ecommerce and digital health platform that would manage protected health information (PHI). Such technology would be required to have compliance with the Health Insurance Portability and Accountability Act (HIPAA) regulations, along with cybersecurity guidelines “Cybersecurity in Medical Devices: Quality System Considerations and Content of Premarket Submissions” published by the FDA. The integration of e-commerce and digital health platforms that utilize AI/LLM technologies can facilitate personalized health communications, but such technologies face challenges such as fairness, bias and trust [342,343].

## 7. Summary and Conclusions

This article describes (1) the home environment as a therapeutic target for the prevention and treatment of chronic diseases and (2) transforming a household goods e-commerce platform into digital health interventions. The “therapeutic home environment” interventions are intended to improve individual and population health by integrating biophilic design, lifestyle medicine, health education, empowerment and consumer behavior. Growing evidence on the health benefits of self-care practices and interactions with natural environments can contribute to the development of precision interventions for the primary and tertiary prevention of chronic diseases, thereby reducing the economic burden and healthcare utilization. In addition to the social impact, we also discuss the broader impact of ecommerce-as-digital-health strategies on (1) improving environmental sustainability and shortening the evidence–practice gap in healthcare and (2) market-driven incentives for consumer brands to invest in creating evidence-based associations between their products and health outcomes for their clients.

The proposed approach significantly differs from existing health promotion methods and digital health platforms. Health promotion strategies such as health education and awareness campaigns traditionally focus on a single and non-personalized message related to public or environmental health, and such campaigns depend on external funding resources and last only for a limited period of time. In contrast, e-commerce as a digital health intervention offers lifelong and personalized health education that is (1) tailored to meet the needs of each individual within a household, (2) embedded in the home environment and daily activities such as online shopping, (3) on-demand, (4) “self-funded” since it is founded from e-commerce revenues and (5) delivered across multiple channels directly to consumers, and through workplaces and healthcare systems. While there are digital health platforms that support hospital-at-home programs, chronic disease management, health coaching and mental wellness, to the best of our knowledge, none of those platforms integrate (1) restorative and multisensory home environments as a part of non-pharmacological interventions or (2) online shopping activities coupled with customized health education. In addition, none of these digital platforms currently offer lifelong prevention (primary and tertiary) of chronic conditions for consumers who are not members of health plans.

This work may encourage lawmakers to increase financial support to advance various aspects of therapeutic home environments (e.g., allocating more research funding to study health outcomes of enriched environments combined with non-pharmacological interventions), or policymakers and urban planners to incentivize creating health-focused residential and commercial spaces. We hope that this perspective may encourage healthcare professionals to include questions about the home environment during motivational interviewing with their patients, while healthcare systems may recognize opportunities to create health-at-home programs for long-term care that would complement their hospital-at-home programs focused on acute-level care. For e-commerce developers, this work spotlights new opportunities to explore the integration of immersive technologies with consumer behavior and health behavior changes [344].

In conclusion, a health-centric household e-commerce platform can provide win–win and scalable solutions to cure chronic diseases while addressing multiple pain points in medicine and healthcare, as exemplified in Table 1.

## Figures and Tables

**Table 1:** Examples of diverse pain points in medicine and the US healthcare related to the prevention and treatment of chronic diseases.

Pain Point	References
50% rates of medication non-adherence; 40% of the most prescribed drugs have black box warning due to adverse effects	[14,15]
Sedentary lifestyle, poor nutrition, chronic stress, loneliness and environmental pollution are major contributors to higher morbidity and mortality	[16,17,18,19,20,21]
The overlooked and underutilized impact of the home environment on patient outcomes and population health	[22,23,24]
The evidence–practice gap (EPG) between published research studies and their implementation to improve patient care	[25,26,27,28]
Inferior US healthcare outcomes, including the healthspan–lifespan gap, compared with other high-income countries	[29,30,31]
Increased mortality and morbidity rates due to medical errors	[32,33]
Public mistrust in medicine and healthcare	[34,35,36]
Healthcare accessibility, affordability and financial toxicity	[37,38]
Healthcare systems incentivize volume instead of value-based care	[39,40]
Decreasing profit margins due to increasing healthcare costs	[41,42]
Market-driven preferences to invest in new medical treatments rather than in primary prevention	[11,12,43,44]
Healthcare utilization impact on environmental pollution and carbon footprint	[45,46,47,48,49]

**Table 2:** Examples of environmental pollution that impact the health of household residents.

Pollution	Source	Health Effects	References
Chemical and biological pollution			
Volatile organic compounds (VOCs) and hydrocarbons	Furniture, building materials	Carcinogenic, reproductive and metabolic defects, dysregulation of cardiovascular functions	[151,152]
Endocrine disruptors	Household products	Increased risks of cancer, obesity, diabetes, infertility, thyroid disease	[153,154]
Dioxin and other carcinogens	Household, personal care products	Increased risks of cancer	[155,156]
Particulate matters (PM) and NO_2_	Industrial pollution, wildfires	Respiratory diseases, increased disability, higher mortality due to cardiovascular disorders and cancer	[157,158,159]
Mycotoxins and mold	Water-damaged indoor spaces	Asthma, chronic inflammation, dysregulation of the immune and nervous systems	[160,161,162,163]
Physical pollution			
Noise	Traffic, industrial noises	Disruption of quality sleep, cardiovascular and immune functions, cognitive impairment	[164,165]
Light	Artificial lighting systems	Disruption of circadian rhythms, quality sleep, increased risk of Alzheimer’s	[166,167]
Radiation	Radon	Lung carcinogen, neuropsychological dysfunctions	[168,169]

**Table 3:** Examples of non-pharmacological modalities integrated into therapeutic home environments coupled with a health-centric household goods e-commerce platform.

Modality	Main Objective	Expected Health Outcomes	Proposed Mechanism of Action	References
Biophilic design and neuro-esthetics	Emulating natural environment and nature-based multisensory experiences to elicit restorative responses, esthetic experiences and comfort	Improving mental health, cardiovascular and cognitive functions; emotional regulation; stress reduction; improving locus of control and engagement	Homeostatic modulation of the autonomic nervous system	[66,101,112,115,119,121,301,302]
Self-care practices and lifestyle medicine	Engaging occupants in health-centric behaviors that lead to clinically meaningful benefits	Improving mental, cardiovascular, metabolic, immune health; reduction of disease symptoms	Anti-inflammatory effects and neuroplasticity	[296,303,304,305]
Patient education	Integrating health literacy and self-efficacy to improve patient decision-making capacity and expectations	Lifestyle-driven disease prevention, reduction of disease symptom, improving disease prognosis	Behavior change and neuroplasticity	[85,86]
Environmental health	Monitoring and mitigating exposure to environmental pollution	Reduction of risk factors for multiple chronic diseases, reduction of disease symptoms	“First, do not harm”, reduction of negative effects of chemical and physical pollution	Listed in Table 2
Conscious shopping	Increasing health-centric consumer behaviors	Considered “inactive ingredient”	Engagement	[306,307,308]

## Data Availability

No new data were created or analyzed in this study. Data sharing is not applicable to this article.

## Supplementary Materials

The following supporting information can be downloaded at: https://www.mdpi.com/article/10.3390/ijerph22020225/s1, Figure S1 shows biophilic refuge space for people living with anxiety. Figure S2 shows a kitchen nook space intended to reduce anxiety symptoms. Figure S3 provides an overview of a typical digital health interventions, including digital therapeutics. Figure S4 illustrates an example of a digital-behavioral-environmental intervention via the e-commerce platform targeting the therapeutic home environment. Figure S5 shows examples of adoption of the household goods e-commerce integrated with digital health platform delivering therapeutic home environments to diverse stakeholders using the B2B2C business model.
