# Supplementary material for: Home Environment as a Therapeutic Target for Prevention and Treatment of Chronic Diseases: Delivering Restorative Living Spaces, Patient Education and Self-Care by Bridging Biophilic Design, E-Commerce and Digital Health Technologies
Source: Int J Environ Res Public Health. 2025 Feb 5;22(2):225. doi: 10.3390/ijerph22020225 (PMC11855921; doi:10.3390/ijerph22020225)

Supplementary Materials

Home Environment as a Therapeutic Target for Prevention and Treatment of Chronic Diseases: Delivering Restorative Living Spaces, Patient Education and Self-care by Bridging Biophilic Design, E-commerce and Digital Health Technologies

Dorothy Huntsman and Grzegorz Bulaj

Supplementary figures:

Figure S1. A biophilic refuge space for people living with anxiety.
Figure S2. A kitchen nook space intended to reduce anxiety symptoms.
Figure S3. An overview of a typical digital health interventions, including digital therapeutics.
Figure S4. An example of a digital-behavioral-environmental intervention via the e-commerce platform targeting the therapeutic home environment.
Figure S5. Examples of adoption of the household goods e-commerce integrated with digital health platform delivering therapeutic home environments to diverse stakeholders using the B2B2C business model.

Figure S1. An example of a biophilic refuge space located in the living room and intended for stress reduction and relief of anxiety symptoms.

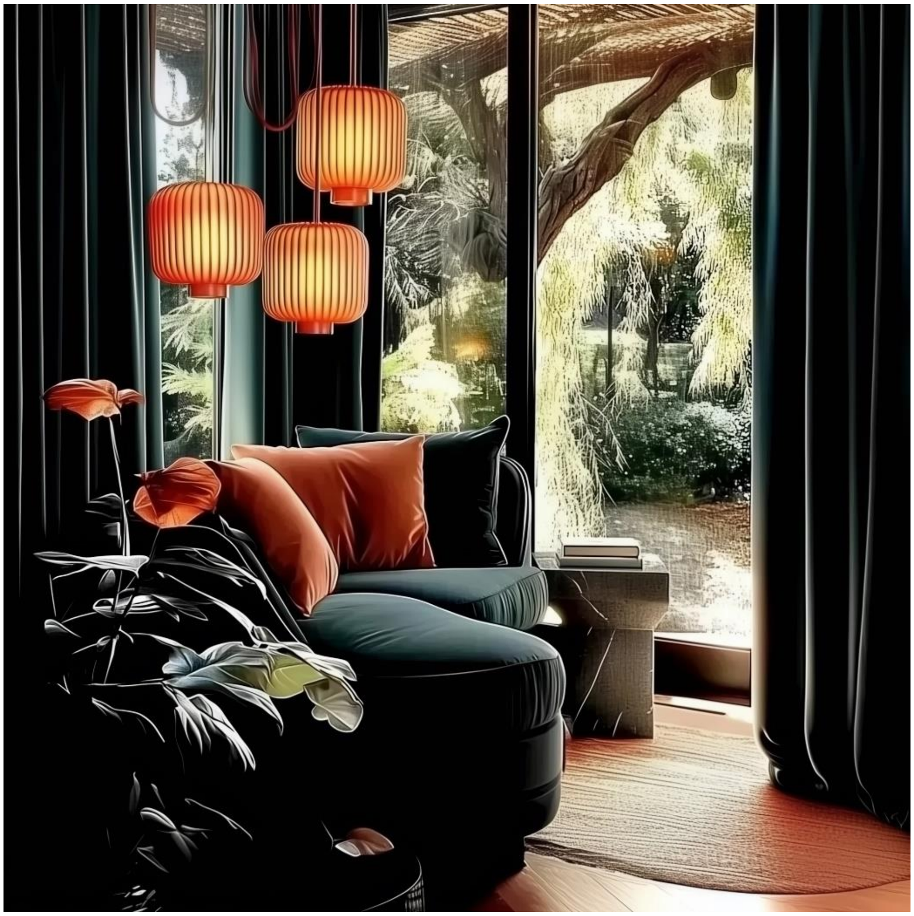

Figure S2. An example of a biophilic refuge space located in the kitchen and intended for stress reduction and relief of anxiety symptoms.

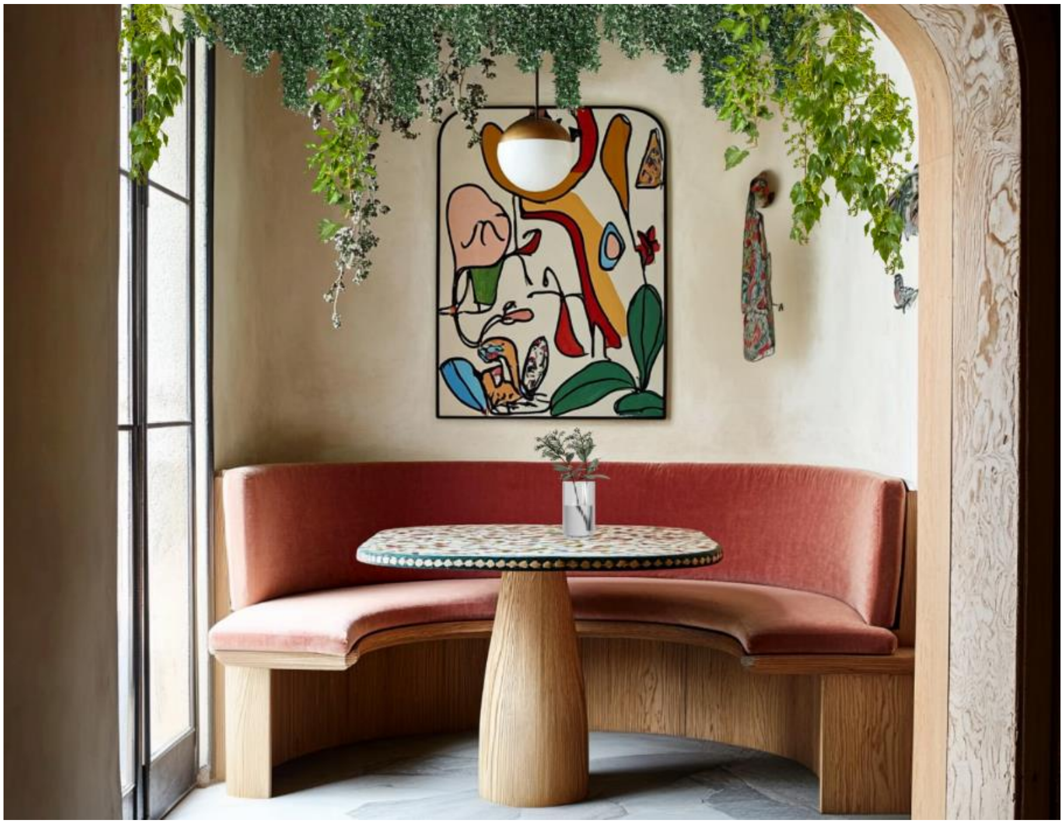

Figure S3. An overview of digital health interventions employing diverse non-pharmacological modalities.

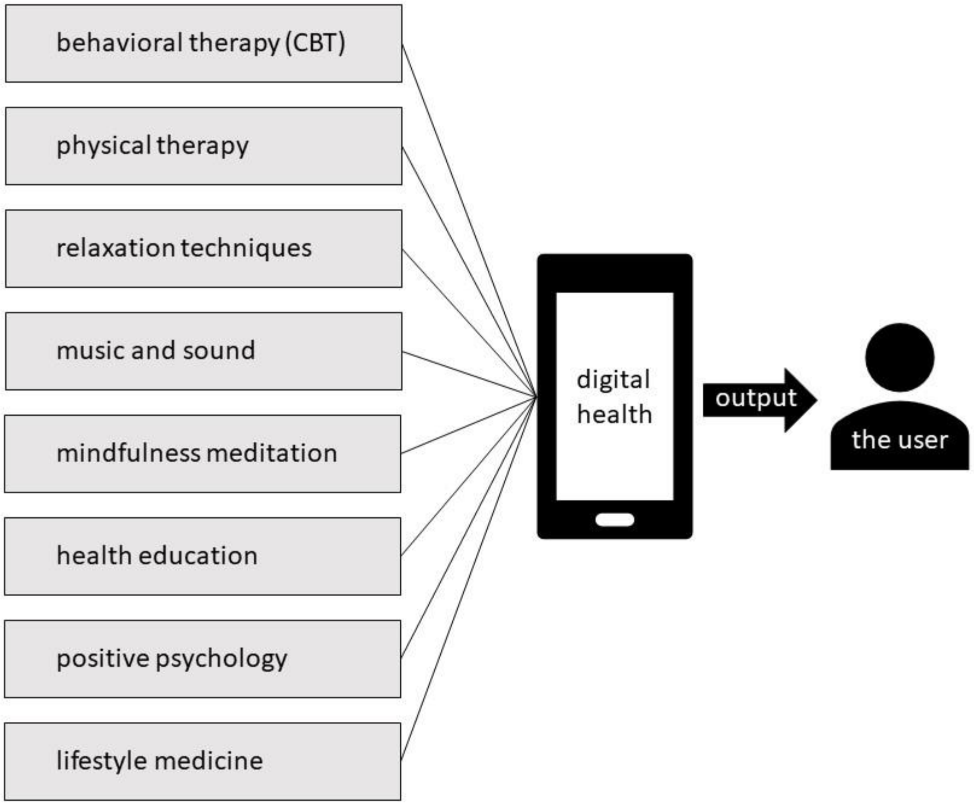

Figure S4. An example of digital-behavioral-environmental interventions using an e-commerce platform delivering the therapeutic home environment.

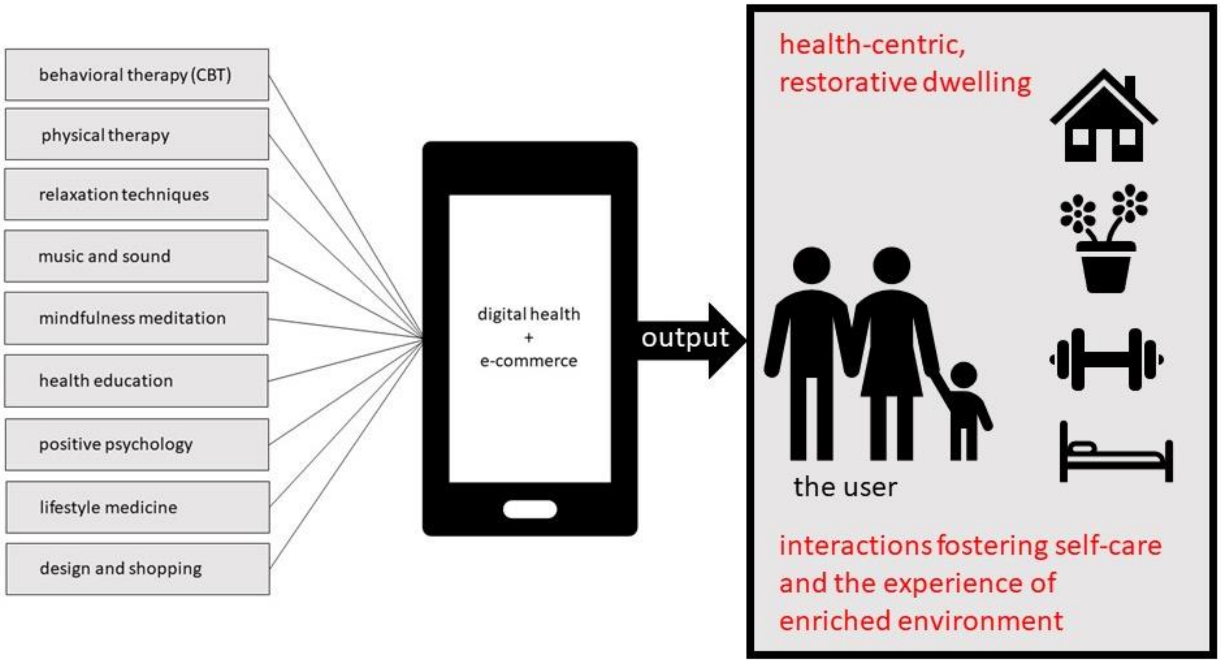

Figure S5. Examples of scaling up adoption of an integrated household goods e-commerce and digital health platform delivering the therapeutic home environments to diverse stakeholders.

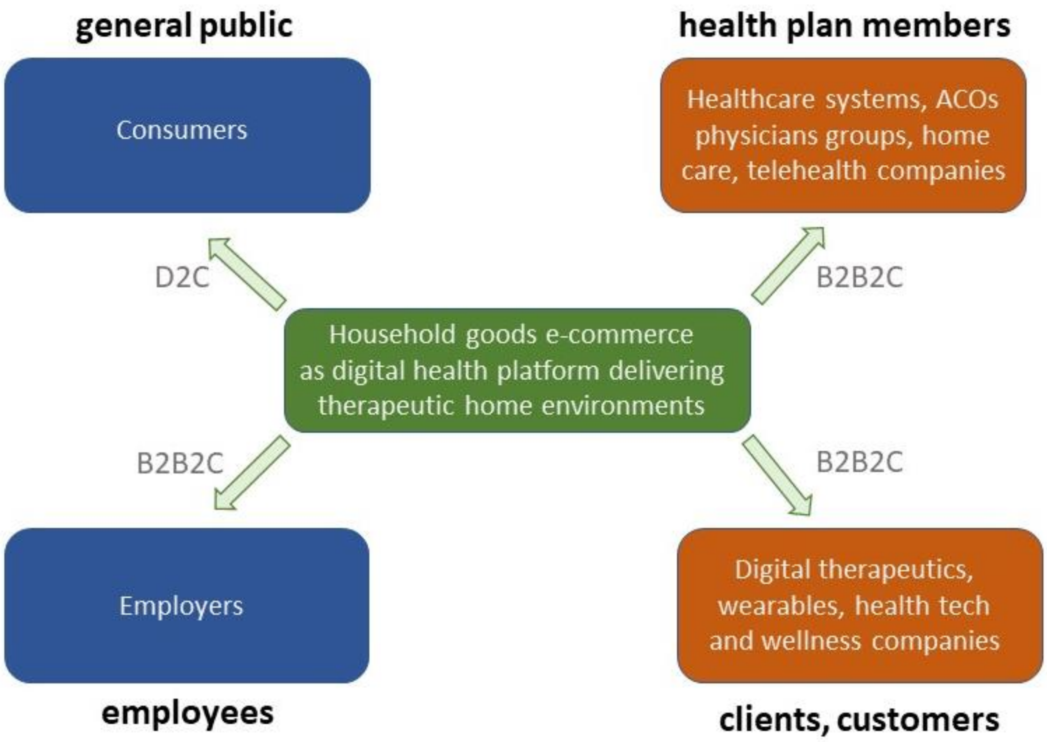

Supplement: Supplementary file 1 [file ijerph-22-00225-s001.zip › ijerph-3417875-supplementary.pdf]
